# A case–control study to investigate association between serum uric acid levels and paroxysmal atrial fibrillation

**DOI:** 10.1038/s41598-022-14622-y

**Published:** 2022-06-20

**Authors:** Xia Zhong, Huachen Jiao, Dongsheng Zhao, Jing Teng

**Affiliations:** 1 Department of First Clinical Medical College, Shandong University of Traditional Chinese Medicine, Jinan, Shandong People’s Republic of China; 2 Department of Cardiology, Affiliated Hospital of Shandong University of Traditional Chinese Medicine, No. 16369, Jingshi Road, Lixia District, Jinan, Shandong People’s Republic of China

**Keywords:** Biomarkers, Diseases, Risk factors

## Abstract

The relationship between serum uric acid (SUA) levels and paroxysmal atrial fibrillation (AF) remains controversial. The objective of this case–control study was to investigate the association between serum SUA levels and paroxysmal AF by gender in 328 patients. This study included 328 hospitalized patients with newly diagnosed paroxysmal AF in China between January 2019 and September 2021. Controls with sinus rhythm were matched (2:1) to cases by age and gender. Baseline data were analyzed using ANOVA, T-test, and Chi-square test. Pearson correlation analyses were used to confirm the correlation between variables, and multivariate regression analyses were used to adjust for covariates. Elevated SUA levels in female patients were significantly associated with paroxysmal AF after adjusting for confounding factors (OR = 1.229, 95% CI 1.058–1.427, *P* = 0.007). Further results showed SUA levels were negatively correlated with high-density lipoprotein cholesterol (HDL-C) (r = − 0.182, *p* = 0.001) and apolipoprotein A1 (APOA1) (r = − 0.109, *p* = 0.049), were positively correlated with low-density lipoprotein cholesterol (LDL-C) (r = 0.169, *p* = 0.002) and prealbumin (PAB) (r = 0.161, *p* = 0.004) . Nevertheless, there was no significant complication difference between SUA levels and paroxysmal AF (*P* > 0.05). Increased SUA in female patients was significantly associated with paroxysmal AF in a Chinese population. This finding implies that it would be interesting to monitor and interfere with hyperuricemia in paroxysmal AF patients.

## Introduction

Atrial fibrillation (AF), an increasingly epidemic and challenging cardiac arrhythmia, is affecting over 33 million individuals worldwide^1,2^. The prevalence of AF is expected to more than double over the next 40 years^3^. AF represents a burgeoning health threat associated with increased risk of stroke, cognitive impairment, systemic embolism, heart failure, and even death^4–8^. In the past decade, despite multifaceted efforts, there has been no significant progress against AF in clinical practice^9^. From a clinical point of view, AF usually begins as paroxysmal, progresses to persistent, and eventually permanent forms of arrhythmias. Unfortunately, paroxysmal AF as the initiator of AF has a single operative success rate of only 66.6% and may be associated with more complications^10,11^. Consequently, the prevention and treatment of paroxysmal AF remain challenging and it is of great significance to investigate serum biomarkers related to the pathological mechanism of paroxysmal AF.

Uric acid is produced by xanthine oxidase (XO) and is the byproduct of purine metabolism in humans^12,13^. Recently, considerable attention has been obtained to the possible mechanism by which hyperuricemia contributes to AF^14,15^. Several studies reported that there was an association between elevated SUA and the risk of AF^16–18^, while results remained an inconclusive measurement, possibly due to differences in the study population, confounding factors, or gender. To our knowledge, studies on different subtypes of relationships between SUA levels and AF are limited, and the potential relationship between paroxysmal AF and SUA levels has not been fully elucidated, especially in the Chinese population.

Therefore, this study aimed to investigate the association between serum SUA levels and paroxysmal AF by gender in 984 patients from China.

## Material and methods

### Study design

This study conducted a retrospective design to investigate 984 hospitalized patients (M/F: 499/485, aged 25–85 years) from the Affiliated Hospital of Shandong University of Traditional Chinese Medicine from January 2019 to September 2021. Using the hospital electronic medical records database, we included the short-term hospitalized community patients with a healthy lifestyle and normal nutritional status, including patients newly diagnosed with paroxysmal AF (M/F:179/149, 67.57 ± 11.16 years) and age-, sex-matched non-AF patients with sinus rhythm. Then, we excluded patients with a medical history of persistent AF and permanent AF, congenital heart disease, valvular disease, heart failure, cardiac surgery, malignancy, current liver or kidney dysfunction, hyperthyroidism, infection, gout, or use of uric acid-lowering drugs, and diuretics, β-blockers, anticoagulants during the study period, as well as pregnant women. Finally, we identified 328 patients with a new diagnosis of paroxysmal AF; a further 656 aged- and sex-matched (2:1) patients with sinus rhythm were randomly extracted as controls. This study was conducted in accordance with the principles of the Helsinki Declaration and approved by the Medical Research Ethics Committee of the Affiliated Hospital of Shandong University of Traditional Chinese Medicine. Because the data are anonymized, the Ethics Committee of Affiliated Hospital of Shandong University of Traditional Chinese Medicine (NO.20200512FA62) agreed to waive informed consent.

### Definition of paroxysmal AF and comorbidities

According to the published guidelines^19^, AF was defined as an arrhythmia lasting for 12-lead ECG to be recorded, or lasting for at least 30 s. Paroxysmal AF was considered spontaneous termination terminated spontaneously or intervention within 7 days of the onset of AF. Hypertension, coronary heart disease, and diabetes were considered comorbidities of paroxysmal AF.

### Definition of SUA

The uricase method was performed to define SUA levels. The conversion standard of SUA levels was defined as 1 mg/dL = 59.48 μmol/L. The SUA levels were divided into three tertiles (men: < 5.01, 5.01− > 6.39, > 6.39; women: < 4.42, 4.42–5.70, > 5.70).

### Screened variables

The baseline information of participants was selected including age, gender, comorbidities (hypertension, coronary heart disease, and diabetes), and serum indicators, such as triglyceride(TG), total cholesterol(TC), low-density lipoprotein cholesterol (LDL-C), high-density lipoprotein cholesterol (HDL-C), apolipoprotein A1(APOA1), apolipoprotein B (APOB), serum creatinine (Scr), aspartate aminotransferase(AST), alanine aminotransferase (ALT), albumin (ALB), prealbumin(PAB), and serum uric acid (SUA).

### Statistical analysis

All statistical analyses were performed using SPSS version 26.0 (SPSS Inc., Chicago, IL, USA) and GraphPad Prism software (version 9.0.0). Data were presented as mean ± SD for continuous variables and as the frequency for categorical variables. The comparison of continuous variables was performed by T-test or ANOVA, and the comparison of categorical variables was performed by Chi-square. The correlation between SUA and metabolic factors was distinguished by Pearson correlation analyses. Multivariate regression analyses were used to adjust for covariates. *P* < 0.05 was considered to be significant and statistical tests were two-sided.

### Ethics approval and consent to participate

Ethics approval was approved by the Ethics Committee of Affiliated Hospital of Shandong University of Traditional Chinese Medicine. Because the data are anonymized, the Ethics Committee of Affiliated Hospital of Shandong University of Traditional Chinese Medicine (NO.20200512FA62) agreed to waive informed consent.

## Results

### Baseline characteristics

As shown in Table 1, 328 paroxysmal AF patients (M/F:179/149, 67.57 ± 11.16 years) and 656 patients with sinus rhythm (M/F:320/336, 68.09 ± 12.28 years) were enrolled. Compared with controls, paroxysmal AF patients were more likely to have hypertension, CHD, and diabetes (*p* < 0.001), significantly higher levels of Scr, AST, and ALT (*P* < 0.05), significantly lower levels of TC, LDL-C, HDL-C, APOA1, APOB, ALB and PAB (*P* < 0.05), and were more likely to use CCBs, ACEI/ARB, and statins (*p* < 0.05). In addition, SUA levels in women with paroxysmal AF patients were significantly higher than controls (*P* < 0.05), while there was no significant difference in men (*P* > 0.05).

**Table 1:** Baseline characteristics in paroxysmal AF patients and controls.

Variable	Controls (n = 656)	Paroxysmal AF patients (n = 328)	*P* value
Age, years	68.09 ± 12.28	67.57 ± 11.16	0.519
**Gender**			0.087
Men	320 (48.78)	179 (54.57)	
Women	336 (51.22)	149 (45.43)	
Hypertension, n (%)	183 (27.90)	191 (58.23)	< 0.001*
CHD, n (%)	106 (16.16)	275 (83.84)	< 0.001*
Diabetes, n (%)	88 (13.41)	103 (31.40)	< 0.001*
TG, mmol/L	1.36 ± 1.31	1.22 ± 1.06	0.072
TC, mmol/L	5.05 ± 1.08	4.20 ± 1.16	< 0.001*
LDL-C, mmol/L	2.98 ± 0.84	2.51 ± 0.97	< 0.001*
HDL-C, mmol/L	1.23 ± 0.33	1.08 ± 0.34	< 0.001*
APOA1, g/L	1.23 ± 0.24	1.11 ± 0.28	< 0.001*
APOB, g/L	0.98 ± 0.24	0.83 ± 0.55	< 0.001*
Scr, μmoI/L	64.82 ± 27.19	76.37 ± 53.45	< 0.001*
AST, U/L	20.64 ± 11.39	24.81 ± 23.35	0.002*
ALT, U/L	20.09 ± 13.82	23.43 ± 23.00	0.016*
ALB, g/L	40.29 ± 4.13	37.49 ± 4.99	< 0.001*
PAB, mg/L	222.79 ± 56.96	190.87 ± 64.99	< 0.001*
SUA, mg/dL	5.20 ± 1.51	5.53 ± 1.99	0.008*
Men	5.78 ± 1.43	5.76 ± 1.95	0.869
Women	4.65 ± 1.37	5.24 ± 2.01	< 0.001*
CCBs, n (%)	73 (11.13)	105 (32.01)	< 0.001*
ACEI/ARB, n (%)	42 (6.40)	187 (57.01)	< 0.001*
Statins, n (%)	98 (14.94)	198 (60.37)	< 0.001*

Data were presented as mean ± SD or n (%).

*paroxysmal AF* paroxysmal atrial fibrillation, *CHD* coronary heart disease, *AST* aspartate aminotransferase, *ALT* alanine aminotransferase, *APOA1* apolipoprotein A1, *APOB* apolipoprotein B, *ALB* albumin, *PAB* prealbumin, *TG* triglyceride, *TC* total cholesterol, *LDL-C* low-density lipoprotein cholesterol, *HDL-C* high-density lipoprotein cholesterol, *SUA* serum uric acid.

*Statistically significant value (*P* < 0.05).

### Association between SUA and paroxysmal AF

Using multivariate logistic regression analysis, the relationship between SUA and paroxysmal AF was investigated. As shown in Table 2, SUA levels were associated with paroxysmal AF after adjusting for hypertension, CHD, diabetes, CCBs, ACEI/ARB, and statins (OR = 1.138, 95% CI 1.018–1.272, *P* = 0.023). After adjusting for age, TG, TC, LDL-C and HDL-C, Scr, AST, ALT, ALB, APOA1, APOB, and PAB, SUA remained significantly correlated with paroxysmal AF (OR = 1.161, 95% CI 1.039–1.298, *P* = 0.008). After further adjustment for all these factors, SUA remained as a significant factor related to paroxysmal AF (OR = 1.229, 95% CI 1.058–1.427, *P* = 0.007). The current results showed the independent association was significant only in women (*P* = 0.003).

**Table 2:** Association between SUA and paroxysmal AF.

	Total		Men		Women	
	OR 95% CI	P value	OR 95% CI	*P* value	OR 95% CI	*P* value
Model1	1.119 (1.035–1.210)	0.005*	0.994 (0.889–1.112)	0.919	1.246 (1.106–1.403)	< 0.001*
Model2	1.138 (1.018–1.272)	0.023*	0.990 (0.843–1.163)	0.906	1.228 (1.040–1.451)	0.016*
Model3	1.161 (1.039–1.298)	0.008*	1.135 (0.954–1.350)	0.152	1.300 (1.118–1.512)	0.001*
Model4	1.229 (1.058–1.427)	0.007*	1.096(0.837–1.435)	0.506	1.419 (1.126–1.787)	0.003*

Model1: crude, no adjustment.

Model2: adjusting for hypertension, CHD and diabetes, CCBs, ACEI/ARB, and statins.

Model3: adjusting for age, TG, TC, LDL-C and HDL-C, Scr, AST, ALT, ALB, APOA1, APOB, and PAB.

Model4: adjusting for all these factors.

*Statistically significant value (*P* < 0.05).

Abbreviations as in Table 1.

### Complication and age differences in the association between SUA levels and paroxysmal AF

**Figure 1:**
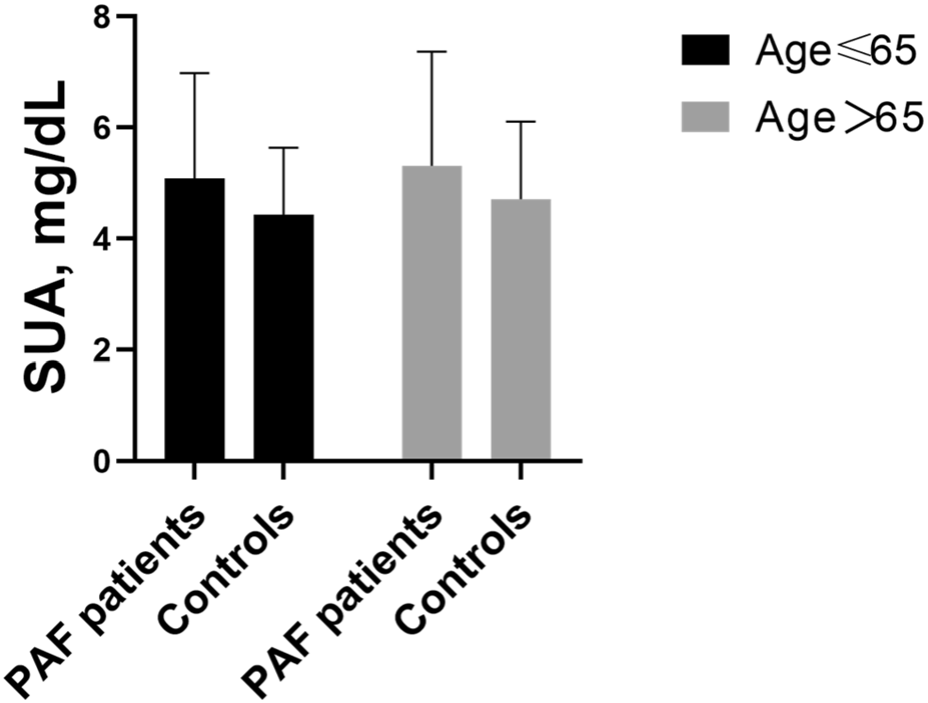
SUA levels in women with paroxysmal AF patients and controls by age. Compared with controls, SUA levels in paroxysmal AF (PAF) patients were significantly higher among women aged ≤ 65 years (5.08 ± 1.90 vs.4.43 ± 1.21 mg/dL, *P* = 0.025) and among women aged > 65 years (5.31 ± 2.06 vs. 4.71 ± 1.40 mg/dL, *P* = 0.003). Abbreviations as in Table 1.

As shown in Table 3, there was no significant complication difference between SUA levels and paroxysmal AF (*P* > 0.05). As shown in Fig. 1, compared with controls, SUA levels in paroxysmal AF patients were significantly higher among women aged ≤ 65 years (5.08 ± 1.90 vs. 4.43 ± 1.21 mg/dL, *P* = 0.025) and among women aged > 65 years (5.31 ± 2.06 vs. 4.71 ± 1.40 mg/dL, *P* = 0.003).

**Table 3:** Complication difference in the association between SUA levels and paroxysmal AF.

Variable	n	SUA (mg/dL)	*P* value
AF + hypertension	191	5.58 ± 1.89	0.613
AF + CHD	275	5.50 ± 1.89	
AF + diabetes	103	5.35 ± 1.95	

Data were presented as mean ± SD.

Abbreviations as in Table 1.

*Statistically significant value (*P* < 0.05).

### Correlation between SUA levels and paroxysmal AF-related metabolic factors

**Figure 2:**
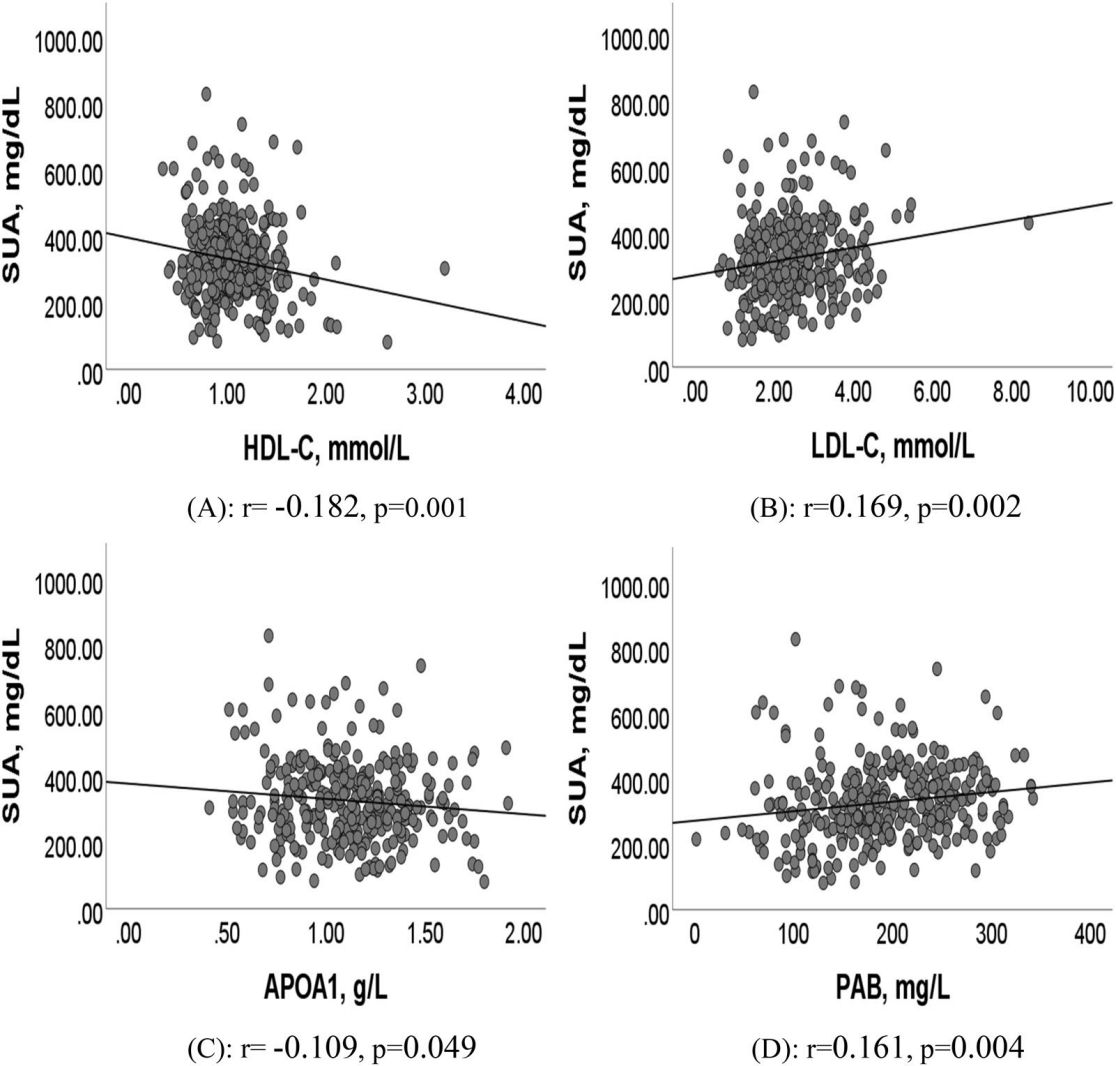
Correlation between SUA levels and metabolic factors in paroxysmal AF patients. (**A**) Correlation between SUA levels and HDL-C in paroxysmal AF patients (r = − 0.182, *p* = 0.001). (**B**) Correlation between SUA levels and LDL-C in paroxysmal AF patients ( r = 0.169, *p* = 0.002). (**C**) Correlation between SUA levels and APOA1 in paroxysmal AF patients (r = − 0.109, *p* = 0.049). (**D**) Correlation between SUA levels and PAB in paroxysmal AF patients (r = 0.161, *p* = 0.004). Abbreviations as in Table 1.

As shown in Fig. 2, SUA levels were negatively correlated with HDL-C (r = − 0.182, *p* = 0.001; Fig. 2A) and APOA1 (r = − 0.109, *p* = 0.049; Fig. 2B), were positively correlated with LDL-C (r = 0.169, *p* = 0.002; Fig. 2A) and PAB (r = 0.161, *p* = 0.004; Fig. 2D).

### Association between SUA levels and metabolic indicators in men and women with paroxysmal AF

As shown in Table 4, we observed higher SUA had higher Scr in men and women (*P* < 0.05). Additionally, the results also showed higher SUA had higher ALB, PAB, TG, and LDL-C in men (*P* < 0.05).

**Table 4:** Association between SUA levels and metabolic factors in paroxysmal AF patients.

Variable	Men (n = 179 )				Women(n = 149 )			
	< 5.01 mg/dL	5.01–6.39 mg/dL	> 6.39 mg/dL	*P* value	< 4.42 mg/dL	4.42–5.70 mg/dL	> 5.70 mg/dL	*P* value
Number, n	58	62	59		45	40	67	
Scr, μmoI/L	68.52 ± 18.98	81.90 ± 16.02	87.27 ± 30.83	< 0.001*	55.47 ± 13.52	67.88 ± 42.05	95.27 ± 119.95	0.037*
AST, U/L	25.41 ± 27.65	26.95 ± 23.65	29.58 ± 36.54	0.744	20.35 ± 9.12	21.75 ± 9.96	23.29 ± 15.14	0.461
ALT, U/L	26.24 ± 29.58	27.55 ± 31.10	27.51 ± 23.67	0.960	17.47 ± 9.41	18.49 ± 11.96	21.08 ± 17.38	0.376
APOA1, g/L	1.03 ± 0.29	1.08 ± 0.27	1.03 ± 0.24	0.494	1.17 ± 0.27	1.20 ± 0.27	1.18 ± 0.30	0.885
APOB, g/L	0.86 ± 1.20	0.78 ± 0.22	0.83 ± 0.25	0.823	0.77 ± 0.20	0.84 ± 0.28	0.89 ± 0.31	0.079
ALB, g/L	35.15 ± 5.31	38.12 ± 5.05	38.38 ± 4.50	< 0.001*	36.94 ± 5.56	38.32 ± 4.09	38.06 ± 4.61	0.349
PAB, mg/L	165.46 ± 67.02	204.34 ± 69.45	206.24 ± 60.01	< 0.001*	174.27 ± 67.74	196.29 ± 50.80	196.35 ± 63.51	0.136
TG, mmol/L	0.92 ± 0.39	1.22 ± 0.78	1.15 ± 0.51	0.016*	1.17 ± 0.60	1.60 ± 2.27	1.27 ± 0.61	0.268
TC, mmol/L	3.72 ± 0.83	4.08 ± 1.02	4.14 ± 1.11	0.050	4.21 ± 1.09	4.58 ± 1.34	4.61 ± 1.35	0.232
LDL-C, mmol/L	2.10 ± 0.63	2.41 ± 0.78	2.64 ± 0.88	< 0.001*	2.49 ± 0.89	2.64 ± 1.10	2.87 ± 1.33	0.221
HDL-C, mmol/L	1.08 ± 0.41	1.02 ± 0.28	0.96 ± 0.22	0.119	1.13 ± 0.33	1.19 ± 0.43	1.11 ± 0.31	0.517

Data were presented as mean ± SD.

Abbreviations as in Table 1.

*Statistically significant value (*P* < 0.05).

## Discussion

This study performed a case–control design to investigate the association between SUA levels and paroxysmal AF by gender in 984 participants from China. We reported that elevated SUA levels in female patients were significantly associated with AF after adjusting for confounding factors. Further results showed that SUA levels were negatively correlated with HDL-C and APOA1, were positively correlated with LDL-C and PAB. However, we did not observe the association between SUA levels and metabolic indicators in women with paroxysmal AF. Meanwhile, there was no significant comorbidity difference between SUA levels and paroxysmal AF. These significant findings might help evaluate the relationship between SUA levels and paroxysmal AF, as well as better understand the pathologic mechanisms of paroxysmal AF.

In the present study, we compared comorbidities and some serum markers between paroxysmal AF patients and sinus rhythm participants. We found that patients with paroxysmal AF were more likely to have hypertension, coronary heart disease, and diabetes, which were important risk factors for paroxysmal AF. Moreover, we observed that TC, LDL-C, and HDL-C were lower in patients with paroxysmal AF. We speculated that this result may be related to lipid-lowering drugs in patients with paroxysmal AF, Wang et al. also obtained similar results with our study^15^. The current results also showed that SUA levels in women with paroxysmal AF patients were significantly higher, but not in men. We further investigated the gender-specific association between SUA levels and paroxysmal AF by multivariate logistic regression analysis. The findings indicated that SUA levels were significantly correlated with paroxysmal AF after adjusting for all confounding factors, while this independent association was only in the total population and women. Although several studies have also reported a strong association between SUA and AF^13,20,21^, few studies have reported the association between SUA levels and paroxysmal AF. A meta-analysis included 31 studies with 504,958 participants that investigated the association between SUA levels and different types of AF, the results suggested that SUA levels were significantly different among participants with new-onset, paroxysmal and persistent AF^22^. Another retrospective study showed that in patients with paroxysmal AF undergoing catheter ablation, increased SUA levels were associated with a higher incidence of AF recurrence^23^. These findings were consistent with our current results.

Evidence about the association between SUA and paroxysmal AF could help understand the multifactorial mechanisms of AF. AF was mediated by inflammation, oxidative stress, neurohormonal activation, and immune activation ^13,24^. Xanthine oxidase can produce SUA, which was upregulated by inflammation and neurohormones. The activation of inflammatory and xanthine stress pathways facilitated by xanthine oxidoreductase were associated with the initiation and maintenance of AF^25,26^. On the one hand, high levels of SUA can mediate the formation of the free radical superoxide anion, and left atrial remodeling is promoted by xanthine stress^27,28^. On the other hand, elevated SUA can induce inflammation by reducing the bioavailability of nitric oxide in the vascular wall^29^. Meanwhile, activation of inflammation also facilitated the production of SUA by augmenting cell destruction^30^. Furthermore, this process may increase the risk factors of developing AF such as hypertension, diabetes, and metabolic syndrome. Therefore, we hope this study will contribute to a better understanding of these important mechanisms of AF.

In addition, our findings also indicated a gender-specific association between SUA and paroxysmal AF, that was, SUA levels were independently associated with paroxysmal AF in women. There are possible mechanisms that could explain the current results. According to previous reports^31,32^, cardiomyocytes have receptors that express sex hormones, and estrogen plays an important role in the occurrence of AF by changing ion channels. Estrogen enhances AF-triggering activity by increasing ICaL and NCX activity. Liang et al.^33^ also found that estrogen prolongs the APD of the atrium and promotes AF by reducing the double-pore potassium channel (TASK-1) in the atrium. Although this finding was consistent with those of previous studies^21,34,35^, some studies still reported inconsistent results. A study based on the general Japanese population found an independent association between SUA and AF in both sexes^36^, and several other studies also reported similar results^37,38^. The gender-specific association between SUA levels and paroxysmal AF remains controversial, and more evidence is needed to confirm this relationship and the underlying mechanisms in the future.

Additionally, we further explored the correlation between SUA levels and paroxysmal AF-related metabolic factors. As far as we know, this is the first study to systematically investigate the relationship between SUA levels and metabolic factors in a paroxysmal AF population. The results of Pearson correlation analysis indicated that SUA levels were negatively correlated with HDL-C and APOA1, and were positively correlated with LDL-C and PAB. There are several possible reasons for the current results. First, it has been demonstrated that ApoA1 levels of paroxysmal AF patients decreased significantly^39^; Second, the anti-inflammatory and antioxidant properties of HDL-C and APOA1 may prevent the formation of the AF matrix and risk factors^40–42^; Third, it more and more appeared that abnormal levels of LDL-C might increase the risk of incident AF^43,44^; In addition, lower serum PAB are associated with inflammatory status, impaired cardiac function, and cardiovascular risk ^45,46^, and it has been found in the AF patients^47^. Nevertheless, we did not observe the association between SUA levels and metabolic indicators in women with paroxysmal AF. Certainly, it is essential to further confirm these relationships and explore their potential mechanisms.

The current study had several obvious limitations that deserve mention. First, the retrospective case–control design couldn’t determine the causality between SUA levels and paroxysmal AF pathology. Therefore, it is essential to confirm the results in prospective cohort studies. Second, smaller sample size was limited to patients visiting a single hospital in China, the results can’t be extrapolated to the general population. Thus, large-sample and multi-center population studies are needed in the future. Third, we didn’t investigate the age-related association between SUA levels and paroxysmal AF. Fourth, due to the limited sample size, we failed to match more relevant factors, such as comorbidities, medications, etc. Additionally, several potential confounding factors such as inflammation and oxidative stress state should also be considered. Nevertheless, this study did provide a new perspective to better understand the pathologic mechanisms of paroxysmal AF. Further prospective studies are needed to confirm the current results and age-specific association between SUA levels and paroxysmal AF.

## Conclusion

In conclusion, we reported herein that elevated SUA in female patients was significantly associated with paroxysmal AF. Current findings proposed the hypothesis that elevated SUA might be a significant serum marker involved in the pathologic progression of paroxysmal AF along with several metabolic factors. Further rigorous investigations were needed to confirm these findings and explore the potential mechanisms.

## Data Availability

The datasets are not publicly available due to them containing information that could compromise research participant privacy, but the minimal data are available from the corresponding author on reasonable request.

## References

[1] Waldmann V (2020). Association between atrial fibrillation and sudden cardiac death: Pathophysiological and epidemiological insights. Circ. Res..

[2] Wijesurendra RS, Casadei B (2019). Mechanisms of atrial fibrillation. Heart.

[3] Krijthe BP (2013). Projections on the number of individuals with atrial fibrillation in the European Union, from 2000 to 2060. Eur. Heart J..

[4] Healey JS, Amit G, Field TS (2020). Atrial fibrillation and stroke: How much atrial fibrillation is enough to cause a stroke?. Curr. Opin. Neurol..

[5] Diener HC, Hart RG, Koudstaal PJ, Lane DA, Lip GYH (2019). Atrial fibrillation and cognitive function: JACC review topic of the week. J. Am. Coll. Cardiol..

[6] Berg DD (2019). Performance of the ABC scores for assessing the risk of stroke or systemic embolism and bleeding in patients with atrial fibrillation in ENGAGE AF-TIMI 48. Circulation.

[7] Santhanakrishnan R (2016). Atrial fibrillation begets heart failure and vice versa: Temporal associations and differences in preserved versus reduced ejection fraction. Circulation.

[8] Lopes RD (2018). Digoxin and mortality in patients with atrial fibrillation. J. Am. Coll. Cardiol..

[9] Lau DH, Nattel S, Kalman JM, Sanders P (2017). Modifiable risk factors and atrial fibrillation. Circulation.

[10] Ganesan AN (2013). Long-term outcomes of catheter ablation of atrial fibrillation: A systematic review and meta-analysis. J. Am. Heart Assoc..

[11] Link MS, Haïssaguerre M, Natale A (2016). Ablation of atrial fibrillation: Patient selection, periprocedural anticoagulation, techniques, and preventive measures after ablation. Circulation.

[12] Tamariz L (2011). Association of serum uric acid with incident atrial fibrillation (from the Atherosclerosis Risk in Communities [ARIC] study). Am. J. Cardiol..

[13] Tamariz L, Hernandez F, Bush A, Palacio A, Hare JM (2014). Association between serum uric acid and atrial fibrillation: A systematic review and meta-analysis. Heart Rhythm..

[14] Kuwabara M (2017). Hyperuricemia is an independent competing risk factor for atrial fibrillation. Int. J. Cardiol..

[15] Wełnicki M (2021). Hyperuricemia as a marker of reduced left ventricular ejection fraction in patients with atrial fibrillation: Results of the POL-AF registry study. J. Clin. Med..

[16] Hidru TH (2020). Does serum uric acid status influence the association between left atrium diameter and atrial fibrillation in hypertension patients?. Front. Cardiovasc. Med..

[17] Wannamethee SG, Papacosta O, Lennon L, Whincup PH (2018). Serum uric acid as a potential marker for heart failure risk in men on antihypertensive treatment: The British Regional Heart Study. Int. J. Cardiol..

[18] Valbusa F (2013). Relation of elevated serum uric acid levels to incidence of atrial fibrillation in patients with type 2 diabetes mellitus. Am. J. Cardiol..

[19] Calkins H (2018). 2017 HRS/EHRA/ECAS/APHRS/SOLAECE expert consensus statement on catheter and surgical ablation of atrial fibrillation. Europace.

[20] Kwon CH, Lee SH, Lee JY, Ryu S, Sung KC (2018). Uric acid and risk of atrial fibrillation in the Korean general population. Circ. J..

[21] Chen Y (2017). Association between serum uric acid and atrial fibrillation: A cross-sectional community-based study in China. BMJ Open.

[22] Wang X (2021). Relationship between serum uric acid levels and different types of atrial fibrillation: An updated meta-analysis. Nutr. Metab. Cardiovasc. Dis..

[23] He XN (2013). Serum uric acid levels correlate with recurrence of paroxysmal atrial fibrillation after catheter ablation. Chin. Med. J. (Engl.).

[24] Friedrichs K, Klinke A, Baldus S (2011). Inflammatory pathways underlying atrial fibrillation. Trends Mol. Med..

[25] Wu SS (2019). Relationships between serum uric acid, malondialdehyde levels, and carotid intima-media thickness in the patients with metabolic syndrome. Oxid. Med. Cell. Longev..

[26] Srivastava A, Kaze AD, McMullan CJ, Isakova T, Waikar SS (2018). Uric acid and the risks of kidney failure and death in individuals with CKD. Am. J. Kidney Dis..

[27] Korantzopoulos P (2007). The role of oxidative stress in the pathogenesis and perpetuation of atrial fibrillation. Int J Cardiol..

[28] Dudley SC (2005). Atrial fibrillation increases production of superoxide by the left atrium and left atrial appendage: Role of the NADPH and xanthine oxidases. Circulation.

[29] Perez-Ruiz F, Becker MA (2015). Inflammation: A possible mechanism for a causative role of hyperuricemia/gout in cardiovascular disease. Curr. Med. Res. Opin..

[30] Manolis AJ (2017). Serum uric acid and atrial fibrillation. Curr. Med. Res. Opin..

[31] Odening KE (2012). Estradiol promotes sudden cardiac death in transgenic long QT type 2 rabbits while progesterone is protective. Heart Rhythm..

[32] Chen G (2011). Regional genomic regulation of cardiac sodium-calcium exchanger by oestrogen. J. Physiol. (Lond.).

[33] Liang B (2014). Genetic variation in the two-pore domain potassium channel, TASK-1, may contribute to an atrial substrate for arrhythmogenesis. J. Mol. Cell. Cardiol..

[34] Suzuki S (2012). Gender-specific relationship between serum uric acid level and atrial fibrillation prevalence. Circ. J..

[35] Lin WD (2019). High prevalence of hyperuricaemia and its impact on non-valvular atrial fibrillation: The cross-sectional Guangzhou (China) Heart Study. BMJ Open.

[36] Kawasoe S (2016). Uric acid level and prevalence of atrial fibrillation in a Japanese general population of 285,882. Circ. J..

[37] Li S (2019). Cohort study of repeated measurements of serum urate and risk of incident atrial fibrillation. J. Am. Heart Assoc..

[38] Nyrnes A (2014). Uric acid is associated with future atrial fibrillation: An 11-year follow-up of 6308 men and women—The Tromso Study. Europace.

[39] Çınar T, Tanık VO, Gürkan K (2020). Comparison of apolipoprotein-A1 levels between paroxysmal atrial fibrillation patients and healthy subjects. J. Cardiovasc. Thorac. Res..

[40] Trieb M (2019). Atrial fibrillation is associated with alterations in HDL function, metabolism, and particle number. Basic Res. Cardiol..

[41] Yang KC, Dudley SC (2013). Oxidative stress and atrial fifibrillation: Finding a missing piece to the puzzle. Circulation.

[42] Velagaleti RS (2009). Relations of lipid concentrations to heart failure incidence: The Framingham Heart Study. Circulation.

[43] Ference BA (2017). Low-density lipoproteins cause atherosclerotic cardiovascular disease. 1. Evidence from genetic, epidemiologic, and clinical studies. A consensus statement from the European Atherosclerosis Society Consensus Panel. Eur. Heart J..

[44] Guan B (2020). Blood lipid profiles and risk of atrial fibrillation: A systematic review and meta-analysis of cohort studies. J. Clin. Lipidol..

[45] Kawano H (2014). Effect of pimobendan in addition to standard therapy for heart failure on prevention of readmission in elderly patients with severe chronic heart failure. Geriatr. Gerontol. Int..

[46] Franco J (2017). Impact of prealbumin on mortality and hospital readmission in patients with acute heart failure. Eur. J. Intern. Med..

[47] Koca M (2020). Impact of atrial fibrillation on frailty and functionality in older adults. Ir. J. Med. Sci..

